# Web-Based Formal Versus Informal Mindfulness Programs for University Students With and Those Without Recent Self-Injury: Randomized Controlled Trial

**DOI:** 10.2196/70011

**Published:** 2025-11-27

**Authors:** Julia Petrovic, Jessica Mettler, Bilun Naz Böke, Maria A Rogers, Chloe A Hamza, Elana Bloom, Lina Di Genova, Vera Romano, Nancy L Heath

**Affiliations:** 1 Department of Educational and Counselling Psychology, McGill University, 3700 McTavish Street (Room 614), Montreal, QC, H3A 1Y2, Canada, 1 514-398-4242; 2 Department of Psychology, Carleton University, Ottawa, ON, Canada; 3 Department of Applied Psychology and Human Development, University of Toronto, Toronto, ON, Canada; 4 Campus Wellness and Support Services, Concordia University, Montreal, QC, Canada; 5 Office of the Deputy Provost, Student Life and Learning, McGill University, Montreal, QC, Canada; 6 Student Wellness Hub, McGill University, Montreal, QC, Canada

**Keywords:** nonsuicidal self-injury, mindfulness, well-being, psychological need satisfaction, university students

## Abstract

**Background:**

Mindfulness-based programming (MBP) is increasingly being implemented within university settings to support students’ mental health, and it typically includes the instruction of formal mindfulness (FM) and informal mindfulness (IM) activities. However, recent evidence suggests that university students with a history of nonsuicidal self-injury (NSSI) may experience challenges in response to FM (eg, physical or psychological discomfort), whereas the flexibility and brevity inherent in IM may be better tolerated.

**Objective:**

This randomized controlled trial compared web-based FM and IM programs to each other and to an inactive control condition in terms of (1) effectiveness and (2) acceptability as a function of NSSI history (NSSI or no NSSI) and time (1 week after the program or 1 month after the program). Indices of effectiveness included dispositional mindfulness, well-being, perceived stress, psychological need satisfaction, emotion regulation styles, and academic engagement.

**Methods:**

Participants were university students with (n=127) and those without (n=100) past-year NSSI engagement. All procedures were conducted online. Once informed consent was obtained and eligibility was confirmed, participants were randomly assigned to 1 of 3 conditions: FM program, IM program, or inactive control condition. One week before the FM and IM programs commenced, all participants completed a baseline survey assessing all indices of effectiveness. Participants assigned to the FM and IM conditions then attended hour-long mindfulness program group sessions once per week over 4 consecutive weeks, while those assigned to the inactive control condition did not complete any study tasks during this time. Mindfulness program sessions were hosted on the videoconference platform Webex. The same survey completed at baseline was completed again 1 week and 1 month following the intervention period, with added acceptability measures for those who took part in the FM and IM programs.

**Results:**

Overall, the results did not differ as a function of NSSI history. A series of 3-way analyses of covariance revealed that both the FM and IM programs were effective at improving dispositional mindfulness (*P*<.001; η_p_^2^=0.07), nonjudging (*P*=.03; η_p_^2^=0.04), describing (*P*=.007; η_p_^2^=0.05), well-being (*P*=.04; η_p_^2^=0.03), and psychological need satisfaction (*P*=.005; η_p_^2^=0.05) immediately after the program, with these improvements sustained 1 month later. Neither program resulted in improved awareness, nonreacting, observing, stress, emotion regulation styles, or academic engagement (all *P*>.05). Moreover, 3-way ANOVAs revealed high acceptability of both the FM and IM programs, with a preference for IM immediately after the program (*P*=.03; η_p_^2^=0.03).

**Conclusions:**

The findings of this study underscore the effectiveness and acceptability of the FM and IM programs to MBP in the university context, as well as the potential value of explicitly teaching and similarly emphasizing both FM and IM, an approach that may be optimally responsive to diverse needs and preferences among students.

## Introduction

### Background

University students often report elevated levels of stress and mental health difficulties, which have been shown to impede their academic performance [1,2]. In response to these trends, universities frequently supplement mental health services with universal resilience-building programming aimed at promoting students’ mental health and coping capacity on a larger scale [3,4]. Mindfulness (ie, the purposeful awareness and nonjudgmental acceptance of one’s present moment experiences [5]) is a common foundation of this programming due to a large body of evidence demonstrating its mental health benefits among general university student samples [6-8].

The aim of mindfulness-based programming (MBP) in the university context is to increase students’ general tendency to be mindful (ie, their dispositional mindfulness) by increasing the frequency with which they experience states of mindfulness, through repeated practice of the strategies taught [9-11]. These strategies typically include a combination of formal mindfulness (FM) and informal mindfulness (IM) activities. FM activities involve focusing one’s attention on thoughts, emotions, and physical sensations for a sustained period of time with nonjudgmental acceptance, often through structured meditation. IM activities, on the other hand, involve incorporating mindfulness into one’s daily activities [12], such as becoming intentionally aware of and nonjudgmental toward one’s sensory experiences during a commute to campus. The emphasis of most MBP approaches is on teaching FM activities and encouraging their regular use, with the assumption that the tendency to engage in IM will naturally follow [13]. However, a limitation of this common approach is that individual differences in responses to FM activities are not considered.

For instance, recent research evidence suggests that university students who have a history of engaging in nonsuicidal self-injury (NSSI) may be predisposed to find certain aspects of standard mindfulness instruction challenging [14]. NSSI is defined as the deliberate and self-inflicted damage of body tissue without suicidal intent and for purposes not socially or culturally sanctioned [15], and as many as 20% of university students report a lived experience of NSSI [16,17]. These students are particularly prone to experiencing challenges in terms of stress, coping, and well-being in the university context [18,19], and therefore stand to benefit greatly from MBP that is adapted to their needs.

University students with a history of NSSI also tend to report elevated levels of emotion regulation difficulties [20], self-criticism [21], alexithymia (ie, an inability to identify and describe one’s emotional experiences [22]), and a complex relationship with their body [23,24], and may report a history of trauma [25]. As a result, these students may encounter challenges with FM activities due to their emphasis on maintaining a prolonged focus on thoughts, emotions, and bodily sensations [14]. In contrast, the brevity and flexibility inherent in IM activities may be well-received. Indeed, findings from a recent qualitative study revealed that while university students with a history of NSSI perceived FM activities to be generally acceptable, they also noted a range of challenges in response to them, including feelings of impatience and frustration around the inability to focus, and perceived the hyperawareness of bodily sensations as aversive or upsetting [26]. Conversely, IM activities were perceived as particularly enjoyable and easy to implement into students’ daily routines.

Although these preliminary findings suggest that the use of IM activities warrants further consideration, particularly among university students with a history of NSSI, there is limited research evidence on the effectiveness of IM activities in isolation. In other words, MBP approaches most often comprise both FM and IM activities, and are evaluated as a whole [6-8], while only a handful of studies have attempted to parse out the benefits of informal practice in the absence of any simultaneously occurring formal practice [27-30]. Findings from these studies suggest that IM practice may have benefits, including decreased stress, anxiety, and depression, as well as increased state and dispositional mindfulness, self-compassion, well-being, positive affect, and life satisfaction. Meanwhile, studies that have evaluated MBP comprising both FM and IM have reported mixed evidence regarding the effectiveness of IM as a standalone practice. Specifically, some of these studies have found FM practice to be more beneficial than IM [31-33], some have found IM practice to be more beneficial than FM [34-36], and others have found no difference between FM and IM in terms of their associated benefits [37].

To our knowledge, only 1 study has experimentally compared the impacts of FM and IM activities in the form of mindfulness inductions (ie, brief, single-session mindfulness practices) among university students with and those without a history of NSSI [38]. Findings from this previous study revealed that only the IM induction was consistently preferred relative to a control task and led to significant improvements in both state stress and state mindfulness, while the FM induction led to significant improvements only in state mindfulness. Taken together, these findings highlight the potential promise of IM activities among university students with and those without a history of NSSI. Nevertheless, the longer-term impacts of routine engagement in these activities, as well as possible differences in these longer-term impacts between students with and those without a history of NSSI, have yet to be empirically investigated.

### This Study

In light of the rising use of MBP in the university context, coupled with early evidence suggesting that certain elements of standard mindfulness instruction may not be well-received by students with a history of NSSI (who comprise roughly one-fifth of the university student body [16,17]), there is a timely need to further investigate the acceptability and effectiveness of FM versus IM programs among university students with and those without a history of NSSI. Thus, the first objective of this study (objective 1) was to investigate potential differences in MBP effectiveness among university students as a function of group (recent NSSI or no NSSI), condition (FM program, IM program, or inactive control), and follow-up time point (postprogram or 1-month follow-up). Of note, effectiveness was inferred via improvements over time in dispositional mindfulness, well-being, perceived stress, psychological need satisfaction, emotion regulation styles, and academic engagement, accounting for baseline levels of these variables. It was expected that all indices of effectiveness would be significantly improved at both follow-up time points among students assigned to the FM and IM conditions relative to those assigned to the inactive control condition (hypothesis 1a). In addition, among those with a recent history of NSSI, the IM program was also expected to be more effective than the FM program (hypothesis 1b). The second objective (objective 2) was to compare the acceptability of FM instruction with that of IM instruction among university students with and those without a recent history of NSSI. Only students with a recent history of NSSI were expected to report greater acceptability in response to IM relative to FM (hypothesis 2).

## Methods

### Participants

A medium effect size was anticipated across outcomes in light of previous findings of web-based MBP generally demonstrating small to medium effects among university students [8,39], coupled with studies that have isolated IM instruction and found it to have medium to large effects [28,30]. Moreover, an attrition rate of 25% was anticipated, given previous reviews of web-based MBP that report synthesized attrition rates of 22% to 24% [40-42]. An a priori power analysis for a 3-way repeated measures ANOVA with a desired power of 0.80, anticipating a medium effect size (Cohen *f*^2^=0.25), and accounting for an attrition rate of 25% revealed a minimum initial sample size of 192 participants. Participants were students from 2 large universities in Canada. Eligibility criteria to participate included (1) active enrollment at 1 of the 2 universities, (2) being at least 18 years old, and (3) having engaged in NSSI on at least 5 separate days within the past 12 months (a frequency/recency criterion consistent with the Diagnostic and Statistical Manual of Mental Disorders, Fifth Edition [DSM-5] diagnostic criteria for NSSI disorder [43]) or reporting never having engaged in NSSI in their lifetime.

### Ethical Considerations

Research ethics board approvals were obtained from the 2 host universities: McGill University (#23-06-016) and Concordia University (#30018739). The first page of the screening questionnaire was the study consent form; participants were prompted to provide their informed consent before being able to proceed to the questions. All study data were deidentified prior to analysis. Upon study completion, participants were provided with online resources containing material on FM and IM for self-directed use and were compensated up to CAD $55 (approximately US $39) via e-transfer, with individual compensation amounts dependent upon the number of surveys completed (baseline: CAD $10 [approximately US $7]; postprogram: CAD $20 [approximately US $14]; 1-month follow-up: CAD $25 [approximately US $18]).

### Procedure

This randomized controlled trial was registered at ClinicalTrials.gov prior to recruitment (ID NCT06038942). All procedures were conducted online. Participants were recruited through electronically distributed flyers (via university listservs and social media pages) and emails sent to an existing database of students who agreed to be contacted for future studies by our research team. Interested students completed a screening questionnaire to determine eligibility. Following this, eligible participants were gender-matched to the greatest extent possible within each group (NSSI or no NSSI) before being randomly assigned by the first author (JP) using a 1:1:1 allocation ratio and a random number generator to 1 of 3 parallel conditions: FM, IM, or inactive control. Following data collection with cohort A, this allocation ratio was adjusted due to attrition within the FM and IM conditions to assign a greater proportion of cohort B students to these 2 conditions relative to the inactive control; this was the only deviation from the registered protocol. Neither JP nor the participants were blinded to these assignments.

One week before the FM and IM instructional programs were scheduled to begin, participants from all 3 conditions completed an online survey assessing their baseline dispositional mindfulness, well-being, perceived stress, psychological need satisfaction, emotion regulation styles, and academic engagement. To access the survey, participants were instructed to click a personalized link that was shared with them via email. Participants were required to complete this survey within a 1-week period and were permitted to skip any items that they were not comfortable responding to.

As of the following week, participants assigned to the FM and IM conditions then attended hour-long mindfulness program sessions in groups of 7 to 15 students (18 groups total) once per week over 4 consecutive weeks, either in fall 2023 (cohort A) or winter 2024 (cohort B). Both cohorts’ programs took place mid-semester (ie, in mid-October/November or mid-February/March), with the 1-month follow-up taking place right before final exams (ie, in early December or early April). These live group sessions were hosted on the video conferencing platform Webex (Cisco). Participants assigned to the inactive control condition did not complete any study tasks during these 4 weeks. The use of an inactive control condition was selected to account for changes in student well-being due to time of the year (eg, seasons, academic demands, midterms, and final exams [44]).

Participants from all 3 conditions completed the same online survey (completed at baseline) at 1 week and 1 month following program completion (ie, postprogram and 1-month follow-up). Participants assigned to the FM and IM conditions also completed measures of program acceptability as part of these surveys. Once again, participants accessed these surveys by clicking a personalized link that was shared with them via email and were required to complete the surveys within a 1-week period.

### Intervention

A detailed outline of the FM and IM program content is provided in Multimedia Appendix 1. All program sessions were led by either the first or second author (having expertise in mindfulness and NSSI research, and being trained in mindfulness-based stress reduction [45,46] and harm reduction in the context of mindfulness and NSSI research). A cofacilitator (ie, an undergraduate or graduate research assistant) was also present at each session to monitor and evaluate the lead facilitator’s implementation fidelity. The FM and IM programs were identical in terms of their psychoeducational content and differed only in terms of the strategies taught. Within both programs, psychoeducational content centered around mindfulness (session 1), stress and emotion regulation (session 2), the role that thoughts play in relation to emotions and behavior (session 3), and the use of self-compassion and a growth mindset to combat self-criticism (session 4). In addition, students in the FM condition were taught four 15-minute guided meditation approaches (ie, body scan, sitting meditation, thought meditation, and loving-kindness meditation) and asked to commit to individual practice once daily, on at least 5 days each week, throughout the 4 weeks of the program. In the IM condition, students were taught how to integrate brief moments of IM (30‐60 s) into their daily routine (eg, by becoming aware of their moment-to-moment sensory experiences) and were guided through a series of demonstrations within the group sessions. These students were asked to commit to practicing IM on at least 5 days each week, as many times as possible on those days, throughout the 4 weeks of the program. At the end of every session, participants were provided with (1) a fillable PDF infographic summarizing that session’s psychoeducational content and providing them with a place to keep track of their home practice and (2) any audio files needed to complete the recommended individual practice.

### Measures

#### Screening Questionnaire

A screening questionnaire was used to confirm students’ eligibility to participate, and it included questions about university enrolment status, age, gender identity, and NSSI history, among other demographic information. Items related to NSSI history included, “Have you ever engaged in nonsuicidal self-injury (eg, self-cutting, self-hitting, burning, bruising, scratching, etc, without suicidal intent)?” Participants who responded “No” were directed to the end of the survey and were included in the no-NSSI group (if the other eligibility criteria were also met). Participants who responded “Yes” were prompted to respond to a follow-up item: “Have you engaged in nonsuicidal self-injury on at least 5 separate days within the past 12 months?” Participants who responded “No” were deemed ineligible to participate, whereas those who responded “Yes” were included in the NSSI group (if the other eligibility criteria were also met).

#### Dispositional Mindfulness (Primary Outcome)

The 39-item Five Facets of Mindfulness Questionnaire (FFMQ) was used to assess dispositional mindfulness globally and in terms of its facets [47]. The FFMQ consists of 5 subscales, each reflecting 1 facet of mindfulness (awareness, nonjudging, nonreacting, observing, and describing). Items are rated on a 5-point Likert scale ranging from 1 (*Never or very rarely true*) to 5 (*Very often or always true*), based on participants’ experiences over the last 2 weeks. A higher global mean reflects greater overall dispositional mindfulness, while a higher subscale mean reflects a greater level of that specific facet of dispositional mindfulness. The 39-item FFMQ demonstrates the highest validity and strongest psychometrics of all adaptations of this measure and is thus recommended for use in intervention studies where pre-post changes in dispositional mindfulness facets are assessed [48]. The overall FFMQ had excellent internal consistency at all time points (*α*=.91, .92, and .93 at baseline, postprogram, and follow-up, respectively). Its individual subscales also demonstrated high internal consistency at all time points (awareness: *α*=.90, .88, and .90; nonjudging: *α*=.93, .94, and .95; nonreactivity: *α*=.82, .86, and .86; observing: *α*=.83, .85, and .86; and describing: *α*=.91, .90, and .91, respectively).

#### Well-Being

The 14-item Warwick-Edinburgh Mental Well-Being Scale (WEMWBS) was used to assess well-being [49]. The WEMWBS is a unidimensional questionnaire with items related to various aspects of well-being, including positive affect, satisfying interpersonal relationships, and positive functioning. Items are rated on a 5-point Likert scale ranging from 1 (*None of the time*) to 5 (*All of the time*), based on the extent to which each statement describes the respondent’s experience over the past 2 weeks. A higher sum score indicates higher well-being. The WEMWBS has demonstrated excellent internal consistency, as well as strong test-retest reliability and convergent validity [49]. It demonstrated excellent internal consistency in this study (*α*=.92, .94, and .94 at baseline, postprogram, and follow-up, respectively).

#### Stress

The 10-item Perceived Stress Scale (PSS-10) was used to assess stress [50]. Respondents rate each item on a 5-point Likert scale, ranging from 1 (*Never*) to 5 (*Very often*), based on how often they felt or thought a certain way over the last 2 weeks. Items include “In the last month, how often have you been upset because of something that happened unexpectedly?” and “In the last two weeks, how often have you felt that you were unable to control the important things in your life?” A higher sum score indicates higher perceived stress. The PSS-10 has demonstrated good internal consistency, test-retest reliability, and convergent validity across several studies [51]. It demonstrated good internal consistency in this study (*α*=.84, .85, and .86 at baseline, postprogram, and follow-up, respectively).

#### Psychological Need Satisfaction

The 24-item Basic Psychological Need Satisfaction and Frustration Scale (BPNSFS) was used to assess satisfaction of the psychological needs for autonomy, relatedness, and competence [52]. The BPNSFS includes six 4-item subscales (autonomy satisfaction, autonomy frustration, relatedness satisfaction, relatedness frustration, competence satisfaction, and competence frustration), although its subscales may also be combined to create a global measure of need satisfaction. Items are rated on a 5-point Likert scale ranging from 1 (*Not at all true*) to 5 (*Completely true*), based on participants’ experiences over the last 2 weeks. Sample items include “I feel capable at what I do” (competence satisfaction), “I feel my choices express who I really am” (autonomy satisfaction), and “I feel excluded from the group I want to belong to” (relatedness frustration). Frustration items were reverse-scored for the purpose of generating a global mean of psychological need satisfaction. The overall BPNSFS demonstrated excellent internal consistency in this study (*α*=.92, .93, and .94 at baseline, postprogram, and follow-up, respectively).

#### Emotion Regulation Styles

The 18-item Emotion Regulation Inventory (ERI) was used to assess emotion regulation styles [53]. The ERI consists of 3 subscales, each pertaining to 1 of 3 emotion regulation styles: integrative (eg, “Sometimes unpleasant emotions (eg, anger, worry, and sadness) help me to understand important things about myself”), suppressive (eg, “In any situation, I prefer not to express my stress or anxiety”), and dysregulated (eg, “It’s difficult for me to control my anxiety or stress”). All items are rated on a 5-point Likert scale ranging from 1 (*Completely disagree*) to 5 (*Completely agree*), based on participants’ experiences over the last 2 weeks. Higher mean scores on the ERI subscales indicate greater levels of that specific emotion regulation style. Research has provided evidence for the factor structure, internal consistency, and validity of this measure and its subscales [53-55]. In addition, the subscales demonstrated high internal consistency at all time points in this study (integrative: *α*=.89, .91, and .92; suppressive: *α*=.88, .87, and .89; and dysregulated: *α*=.87, .88, and .85, respectively).

#### Academic Engagement* *

The 9-item Utrecht Work Engagement Scale for Students (UWES-S) was used to assess academic engagement [56]. The UWES-S is the student version of the most widely used measure to assess work engagement, the Utrecht Work Engagement Scale [57,58]. The UWES-S assesses 3 aspects of academic engagement: vigor (eg, “I feel energetic and capable when I’m studying or going to class”), dedication (“I am proud of my studies”), and absorption (“I feel happy when I am studying intensely”). Items are rated on a 7-point Likert scale ranging from 1 (*Never*) to 7 (*Always/every day*), based on participants’ experiences over the last 2 weeks. A global mean may be computed whereby a higher score reflects greater academic engagement. Research has provided evidence for the factor structure, internal consistency, and validity of the UWES-S and its subscales [56,59]. The overall UWES-S demonstrated excellent internal consistency in this study (*α*=.91, .90, and .92 at baseline, postprogram, and follow-up, respectively).

#### Acceptability

The Theoretical Framework of Acceptability (TFA) questionnaire was used to assess acceptability [60]. This measure assesses the 7 components of the TFA (affective attitude, burden, ethicality, perceived effectiveness, intervention coherence, self-efficacy, and opportunity costs [61]), is adaptable, and can be used to evaluate mental health interventions. It consists of 7 items, each pertaining to 1 of the components listed above, as well as an eighth item that assesses general acceptability. For the purposes of this study, participants indicated the extent to which each statement accurately reflected their experience of the mindfulness program they participated in using a 5-point Likert scale ranging from 1 (*Strongly disagree*) to 5 (*Strongly agree*). Sample items included “I found that this mindfulness program improved my well-being” (perceived effectiveness) and “I found that completing this mindfulness program interfered with my other priorities” (opportunity costs). A global mean was calculated, whereby a higher score indicates greater acceptability. The TFA questionnaire has been similarly adapted and applied in the context of experimental research evaluating a mindfulness intervention, wherein this measure’s reliability and single-factor structure were supported [38]. The TFA demonstrated acceptable internal consistency in this study (*α*=.74 postprogram and at the 1-month follow-up).

The Intrinsic Motivation Inventory (IMI) [62], which is intended to assess participants’ subjective experience of a target activity in experimental research, was also used to assess acceptability from a self-determination theory (SDT) perspective. For the purposes of this study, 4 subscales of this measure were included: interest/enjoyment (5 items; eg, “I thought this mindfulness program was quite enjoyable”), perceived competence (5 items; eg, “I was satisfied with my ability to complete this program’s mindfulness activities”), perceived autonomy (6 items; eg, “I felt like it was not own choice to do this program’s mindfulness activities in the way that I did”), and value/usefulness (7 items; eg, “I would be willing to do this program’s mindfulness activities again because they have some value to me”). All items are rated on a 7-point Likert scale ranging from 1 (*Not at all true*) to 7 (*Very true*), and a higher global mean score indicates greater acceptability. The IMI has been validated across numerous experimental studies, including a mindfulness intervention study, and has demonstrated high reliability and strong convergent validity [63,64]. A global mean comprising the same 4 subscales included in this study has been previously used to evaluate mindfulness interventions, and both the single-factor structure and reliability of this approach were supported [38]. The overall IMI demonstrated excellent internal consistency in this study (*α*=.94 and .93 postprogram and at the 1-month follow-up, respectively).

#### Home Practice Frequency

As participants were instructed to complete home practice of the strategies taught in the week that followed each FM and IM program session, home practice frequency was assessed at the beginning of the second, third, and fourth or final program sessions, and as part of the postprogram survey. It was assessed using a researcher-developed question that asked participants, “Over the last week, on how many separate days did you practice formal mindfulness?” (adapted depending on whether they were in the FM or IM condition). Response options included (1) *Never*, (2) *1‐2 days*, (3) *3‐4 days*, (4) *5‐6 days*, and (5) *Every day*. Responses were recoded into 3 categories for data analysis, merging response options (1) and (2) to denote low use, keeping (3) to denote moderate use, and merging response options (4) and (5) to denote high use.

### Data Analysis Plan

#### Participants

A series of 1-way ANOVAs and chi-square tests were conducted to compare intervention completers to noncompleters in terms of demographic variables (ie, age, gender identity, sexual orientation, and ethnicity), all outcome variables at baseline, NSSI history, and assigned condition.

#### Preliminary Analyses

To confirm condition equivalency (ie, successful randomization), a series of 1-way ANOVAs using baseline scores were conducted (1 for each index of effectiveness). In addition, a series of chi-square tests were conducted to determine whether there were any differences as a function of group (recent NSSI or no NSSI) or condition (FM, IM, or inactive control) in home practice frequency at each time point that it was assessed.

Before proceeding with the primary analyses, correlations were conducted between home practice frequency at each program week (weeks 1, 2, 3, and 4) and change scores (baseline to postprogram) for each index of effectiveness, for the FM and IM conditions separately. This was done to assess whether home practice frequency was significantly associated with changes over time in our indices of effectiveness and to inform whether it should be included as a covariate in the primary analyses. The original continuous scores of home practice frequency were used (ie, before they were transformed into low, moderate, and high categories for the chi-square tests, as described in the Measures section). Bonferroni correction was applied to account for the large number of correlations conducted (ie, .05/104=.0005).

#### Assumption Checking

As a final step prior to conducting the primary analyses (ie, ANOVAs and analyses of covariance [ANCOVAs]), the assumptions of homogeneity of variances, sphericity, and normality were checked. The assumption of homogeneity of variances was checked using the Levene test, while the assumption of sphericity was checked using the Mauchly test. Normality was checked using skewness and kurtosis values divided by their respective standard errors; the resulting *z*-scores were compared to a cutoff value of 2.58 (corresponding to *P*=.01).

#### Primary Analyses: Effectiveness of FM Versus IM Programs (Objective 1)

A series of 3-way (group*condition*time) ANCOVAs were conducted to compare effectiveness across groups (recent NSSI or no NSSI), conditions (FM, IM, or inactive control), and follow-up time points (postprogram or 1-month follow-up) for each index of effectiveness, with the baseline score on that index included as a covariate. Specifically, separate ANCOVAs were conducted for overall dispositional mindfulness, each of the 5 facets of dispositional mindfulness, well-being, perceived stress, psychological need satisfaction, each of the 3 emotion regulation styles, and academic engagement. Simple main effects analyses were conducted to probe significant interactions, and pairwise comparisons were conducted to determine where group differences were present, with and without Bonferroni correction to account for family-wise error.

#### Primary Analyses: Acceptability of FM Versus IM Programs (Objective 2)

Two mixed-design 3-way (group*condition*time) ANOVAs were conducted to compare acceptability across groups (recent NSSI or no NSSI), conditions (FM or IM), and follow-up time points (postprogram or 1-month follow-up) (1 for each measure of acceptability). Simple main effects analyses were conducted to probe significant interactions, and pairwise comparisons were conducted to determine where group differences were present, with and without Bonferroni correction to account for family-wise error.

For both primary analyses (objectives 1 and 2), results are reported without Bonferroni correction, as the patterns of significance seldom differed as a function of whether the correction was applied. Where patterns did differ as a function of the correction, both results are reported.

## Results

### Participants

**Figure 1.**
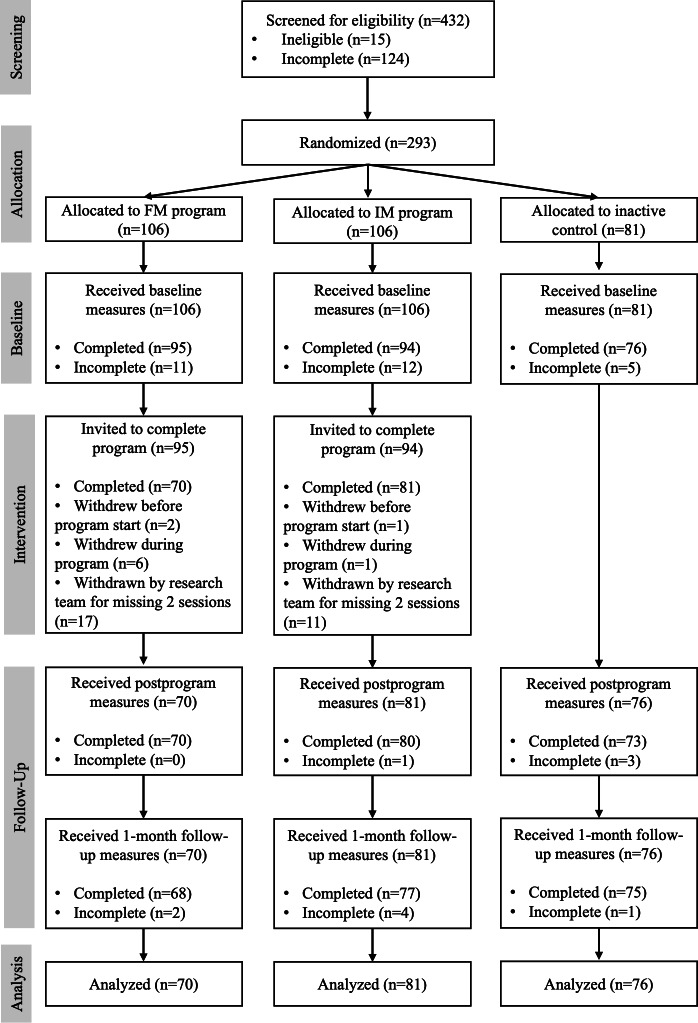
Participant flow diagram. FM: formal mindfulness; IM: informal mindfulness.

A total of 293 university students met the eligibility criteria and were invited to participate in this study. However, 66 (22.5%) were lost to attrition (see Figure 1 for a participant flow diagram). Attrition analyses comparing completers to noncompleters on demographic variables (ie, age, gender identity, sexual orientation, and ethnicity), all outcome variables at baseline, NSSI history, and assigned condition revealed no significant differences, except in terms of assigned condition, whereby those assigned to the inactive control condition were significantly more likely to have completed the study than those assigned to the FM or IM condition. The final sample thus consisted of 227 university students (mean age 22.10, SD 3.37 years; 80.6% [183/227] women), with 127 students reporting a history of NSSI on at least 5 separate days within the past 12 months and 100 reporting never having engaged in NSSI. Participants were randomly assigned to 1 of 3 conditions (FM program, IM program, or inactive control) based on their NSSI history (NSSI or no NSSI). As such, 6 clusters of participants were created. The demographic information for all clusters is provided in Table 1. The study checklist is provided in Multimedia Appendix 2.

**Table 1.** Participant demographics (N=227).

Group	NSSI^a^						No NSSI					
	Formal (n=41)		Informal (n=50)		Control (n=36)		Formal (n=29)		Informal (n=31)		Control (n=40)	
Gender identity, n (%)												
Woman	31 (76)		37 (74)		27 (75)		25 (86)		29 (94)		34 (85)	
Man	5 (12)		7 (14)		7 (19)		4 (14)		2 (7)		5 (13)	
Nonbinary, gender fluid, or 2-spirit	4 (10)		4 (8)		1 (3)		0 (0)		0 (0)		0 (0)	
Trans man or trans woman	1 (2)		2 (4)		1 (3)		0 (0)		0 (0)		1 (3)	
Sexual orientation, n (%)												
Heterosexual	17 (42)		23 (46)		15 (42)		18 (62)		27 (87)		27 (68)	
Gay	3 (7)		2 (4)		0 (0)		0 (0)		0 (0)		2 (5)	
Lesbian	0 (0)		1 (2)		3 (8)		1 (3)		0 (0)		2 (5)	
Bisexual	13 (32)		16 (32)		11 (31)		6 (20)		3 (10)		5 (13)	
Asexual	4 (10)		0 (0)		3 (8)		1 (3)		0 (0)		0 (0)	
Questioning	3 (7)		2 (4)		2 (6)		2 (7)		0 (0)		2 (5)	
Other	1 (2)		5 (10)		2 (6)		0 (0)		1 (3)		1 (3)	
Population group, n (%)												
Arab	4 (10)		0 (0)		3 (8)		1 (3)		1 (3)		1 (3)	
Black	1 (2)		2 (4)		1 (3)		2 (7)		2 (7)		1 (3)	
Chinese, Japanese, or Korean	7 (17)		6 (12)		4 (11)		7 (24)		8 (26)		8 (20)	
Latin American	0 (0)		2 (4)		3 (8)		2 (7)		1 (3)		2 (5)	
South Asian (eg, Indian and Pakistani)	1 (2)		3 (6)		4 (11)		5 (17)		0 (0)		3 (8)	
Southeast Asian (eg, Thai and Filipino)	2 (5)		1 (2)		1 (3)		0 (0)		0 (0)		0 (0)	
West Asian (eg, Iranian and Afghan)	2 (5)		3 (6)		0 (0)		0 (0)		1 (3)		4 (10)	
White	19 (46)		27 (54)		18 (50)		10 (35)		15 (48)		17 (43)	
Other	5 (12)		6 (12)		2 (6)		2 (7)		3 (10)		3 (8)	
Age, mean (SD)	21.88 (3.97)		22.96 (4.05)		21.39 (1.83)		21.66 (3.35)		21.35 (2.60)		22.80 (3.18)	

a NSSI: nonsuicidal self-injury.

### Preliminary Analyses

**Figure 2.**
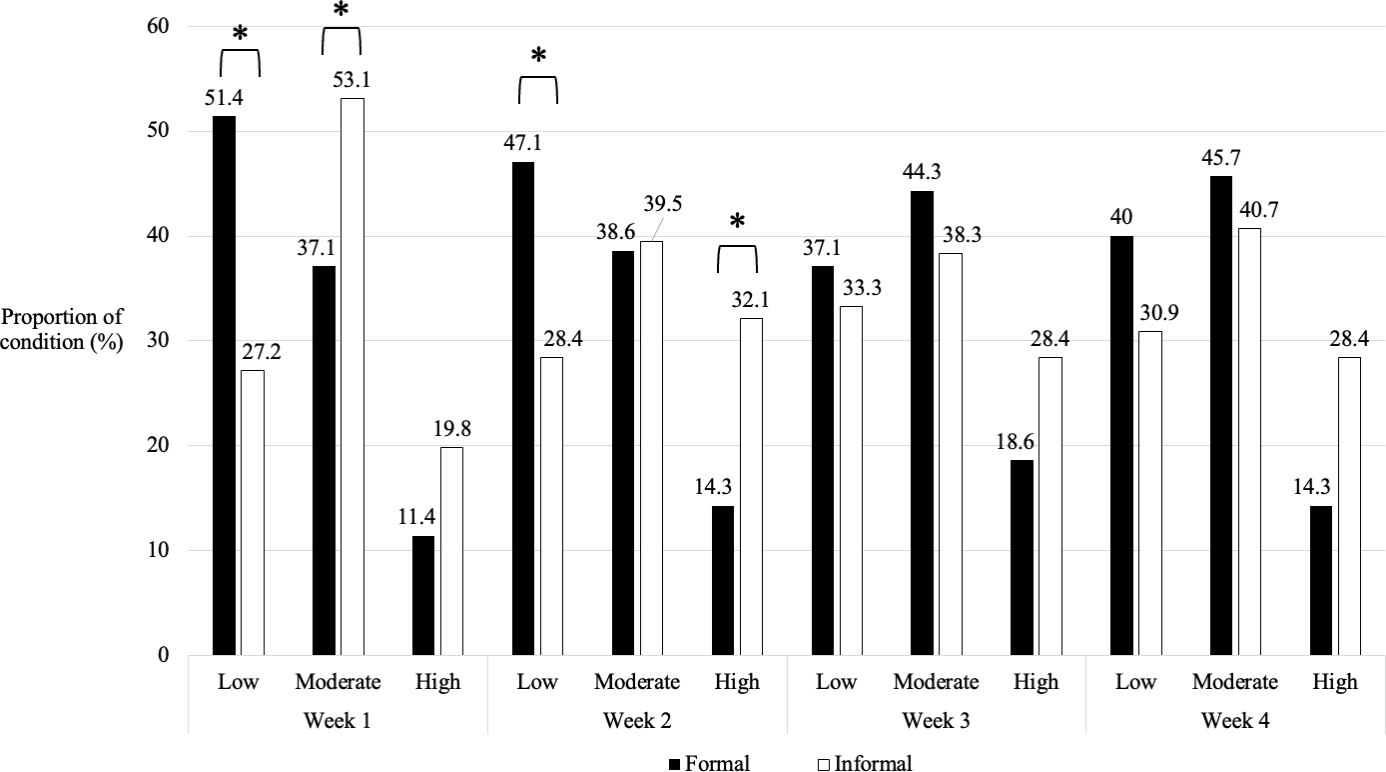
Degree of home practice frequency across conditions by program week. *P<.05.

Results of 1-way ANOVAs revealed no significant differences at baseline in any of the indices of effectiveness as a function of condition (all *P*>.05), thereby confirming condition equivalency and successful randomization. Furthermore, the results of the chi-square tests revealed no significant differences as a function of group (recent NSSI or no NSSI) in terms of home practice frequency during any of the 4 weeks of the FM and IM programs. However, there were significant differences as a function of condition (FM or IM) during the first 2 weeks of the programs (Figure 2). During the first week, those assigned to the FM condition were more likely to report low home practice frequency, whereas those assigned to the IM condition were more likely to report moderate home practice frequency (n=151; *χ*^2^_2_=9.48; *P*=.009). During the second week, those assigned to the FM condition were more likely to report low home practice frequency, whereas those assigned to the IM condition were more likely to report high home practice frequency (n=151; *χ*^2^_2_=8.57; *P*=.01). During the third and fourth weeks, there were no significant differences between those assigned to the FM condition and those assigned to the IM condition in terms of home practice frequency.

The correlations between home practice frequency at each time point and change scores (baseline to postprogram) for each index of effectiveness, which were assessed within the FM and IM conditions separately, were all nonsignificant after applying Bonferroni correction. As such, it was determined that home practice frequency was not significantly associated with changes over time in our indices of effectiveness, and it was therefore not included as a covariate in the primary analyses.

### Assumption Checking

The assumption of homogeneity of variances was met for all outcomes at all time points. Sphericity was only met for suppressive emotion regulation and acceptability (when measured using the TFA). For all other outcomes, this assumption was violated, and a Greenhouse-Geisser correction was applied. Lastly, the assumption of normality was met for every group*condition*time combination across all outcomes, with only 6 exceptions. However, we opted to proceed with the analyses without transforming the data, as ANOVAs and ANCOVAs are generally robust to violations of normality (in addition to being robust to unequal group sizes, as was the case with our data) [65,66].

### Primary Analyses: Effectiveness of FM Versus IM Programs (Objective 1)

Across all indices of effectiveness, there were no significant group*condition*time, group*condition, group*time, or condition*time interactions (all *P*>.05). The only exception was a significant group*time interaction for integrative emotion regulation (*P*=.03), whereby university students with a history of NSSI reported greater integrative emotion regulation than those without such a history at the 1-month follow-up, regardless of condition (mean difference [MD]=0.21; SE=0.10; *P*=.03). All ANCOVA results are reported in detail in Multimedia Appendix 3. Overall, hypothesis 1b was refuted, as the impact of condition (ie, IM, FM, or inactive control) on MBP effectiveness did not differ as a function of NSSI history.

However, in partial support of hypothesis 1a, the main effect of condition was significant for overall dispositional mindfulness (*P*<.001), nonjudging (*P*=.03), describing (*P*=.007), well-being (*P*=.04), and need satisfaction (*P*=.005). Without Bonferroni correction, all of these effects favored both the FM and IM programs over the inactive control condition. When the correction was applied, changes to these patterns were as follows: only FM was favored over the control in terms of nonjudging, only IM was favored over the control in terms of describing, and no pairwise comparisons were significant in terms of well-being. Detailed results pertaining to the main effect of condition for all indices of effectiveness are presented in Tables2  3, given their relevance to our first objective.

**Table 2.** Baseline observed means and combined postprogram and follow-up marginal means across effectiveness outcomes and conditions.

Variable and condition	Baseline observed value, mean (SD)	Combined postprogram and follow-up marginal value, mean (SE)
Overall dispositional mindfulness^a^		
FM^b^	2.87 (0.48)	3.11 (0.04)
IM^c^	2.84 (0.54)	3.12 (0.04)
Control	2.91 (0.55)	2.92 (0.04)
Awareness		
FM	2.69 (0.78)	2.88 (0.06)
IM	2.58 (0.86)	2.82 (0.06)
Control	2.74 (0.81)	2.67 (0.06)
Nonjudging^a^		
FM	2.89 (1.07)	3.28 (0.08)
IM	2.92 (0.96)	3.22 (0.08)
Control	2.85 (0.91)	2.98 (0.08)
Nonreacting		
FM	2.68 (0.64)	2.85 (0.07)
IM	2.62 (0.67)	2.90 (0.06)
Control	2.73 (0.69)	2.71 (0.06)
Observing		
FM	3.12 (0.80)	3.31 (0.07)
IM	3.04 (0.85)	3.36 (0.06)
Control	3.15 (0.72)	3.20 (0.06)
Describing^a^		
FM	2.96 (0.85)	3.21 (0.06)
IM	3.00 (0.91)	3.27 (0.05)
Control	3.04 (0.86)	3.03 (0.06)
Well-being^a^		
FM	41.14 (9.73)	45.34 (0.84)
IM	42.33 (8.81)	45.49 (0.79)
Control	41.63 (9.99)	42.92 (0.80)
Stress		
FM	22.76 (6.49)	19.82 (0.56)
IM	22.07 (6.00)	20.64 (0.53)
Control	22.93 (6.49)	21.60 (0.54)
Psychological need satisfaction^a^		
FM	3.28 (0.60)	3.46 (0.05)
IM	3.34 (0.62)	3.49 (0.05)
Control	3.26 (0.65)	3.27 (0.05)
Integrative ER^d^		
FM	3.43 (1.01)	3.87 (0.07)
IM	3.60 (0.93)	3.84 (0.07)
Control	3.69 (0.77)	3.68 (0.07)
Suppressive ER		
FM	2.96 (1.06)	3.01 (0.08)
IM	3.16 (1.10)	3.00 (0.08)
Control	3.08 (0.83)	3.04 (0.08)
Dysregulated ER		
FM	3.04 (1.03)	3.00 (0.08)
IM	3.20 (0.99)	2.92 (0.08)
Control	3.06 (0.85)	3.05 (0.08)
Academic engagement		
FM	3.13 (1.07)	3.16 (0.08)
IM	2.91 (1.17)	3.11 (0.08)
Control	2.95 (0.92)	2.92 (0.08)

a Significant main effect of condition.

b FM: formal mindfulness.

c IM: informal mindfulness.

d ER: emotion regulation.

**Table 3.** ANCOVA^a^ results for the main effect of condition across effectiveness outcomes.

Variable	ANCOVA results (main effect of condition)			Pairwise comparison results^b^
	*F* test (*df*)	*P* value	η_p_^2^	
Overall dispositional mindfulness^c^	7.60 (2, 205)	<.001	0.07	FM^d^-IM^e^: MD^f^=−0.01; SE=0.06; *P*=.80 FM-Control^c^: MD=0.19; SE=0.06; *P*=.002 IM-Control^c^: MD=0.20; SE=0.06; *P*<.001
Awareness	3.04 (2, 205)	.05	0.03	—^g^
Nonjudging^c^	3.75 (2, 205)	.03	0.04	FM-IM: MD=0.06; SE=0.12; *P*=.58 FM-Control^c^: MD=0.30; SE=0.12; *P*=.01 IM-Control^c^: MD=0.24; SE=0.12; *P*=.04
Nonreacting	2.53 (2, 205)	.08	0.02	—^g^
Observing	1.66 (2, 205)	.19	0.02	—^g^
Describing^c^	5.15 (2, 205)	.007	0.05	FM-IM: MD=−0.06; SE=0.08; *P*=.45 FM-Control^c^: MD=0.18; SE=0.08; *P*=.02 IM-Control^c^: MD=0.24; SE=0.08; *P*=.002
Well-being^c^	3.22 (2, 209)	.04	0.03	FM-IM: MD=−0.15; SE=1.15; *P*=.90 FM-Control^c^: MD=2.42; SE=1.16; *P*=.04 IM-Control^c^: MD=2.57; SE=1.13; *P*=.02
Stress	2.60 (2, 209)	.08	0.02	—^g^
Psychological need satisfaction^c^	5.43 (2, 207)	.005	0.05	FM-IM: MD=−0.03; SE=0.07; *P*=.71 FM-Control^c^: MD=0.19; SE=0.07; *P*=.01 IM-Control^c^: MD=0.21; SE=0.07; *P*=.003
Integrative ER^h^	1.92 (2, 208)	.15	0.02	—^g^
Suppressive ER	0.10 (2, 208)	.91	0.00	—^g^
Dysregulated ER	0.72 (2, 208)	.49	0.01	—^g^
Academic engagement	2.78 (2, 209)	.07	0.03	—^g^

a ANCOVA: analysis of covariance.

b Pairwise comparisons are reported without Bonferroni correction; a positive mean difference favors the first condition noted.

c Significant finding.

d FM: formal mindfulness.

e IM: informal mindfulness.

f MD: mean difference.

g Not significant.

h ER: emotion regulation.

In addition, there was a significant main effect of group on well-being (*P*=.001), need satisfaction (*P*=.02), stress (*P*=.004), and academic engagement (*P*=.02), such that university students with a recent history of NSSI reported lower well-being, need satisfaction, and academic engagement, and higher stress, relative to those without such a history (regardless of condition or follow-up time point; see Multimedia Appendix 3). Finally, there was a significant main effect of follow-up time point on well-being (*P*=.04) and stress (*P*=.02), although their respective pairwise comparisons were not significant (see Multimedia Appendix 3).

### Primary Analyses: Acceptability of FM Versus IM Programs (Objective 2)

**Figure 3.**
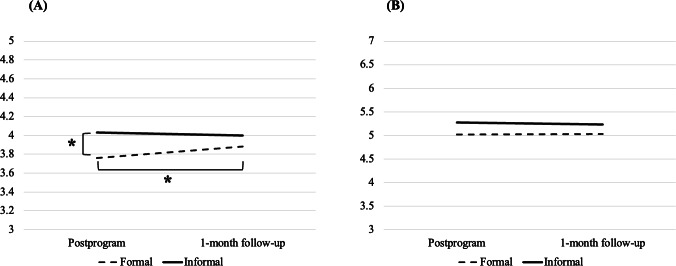
Estimated marginal means of acceptability by measure, condition, and time. (A) Theoretical Framework of Acceptability questionnaire (possible range: 1-7); (B) Intrinsic Motivation Inventory (possible range: 1-5). *P<.05.

All ANOVA results are reported in detail in Multimedia Appendix 3 and displayed graphically in Figure 3. When acceptability was assessed using the TFA questionnaire, there were no significant group*condition*time, group*condition, or group*time interactions (all *P*>.05). However, there was a significant condition*time interaction (*P*=.02). Simple main effects analyses revealed a significant simple main effect of condition at time 1 only, whereby acceptability was higher in response to the IM condition than the FM condition (MD=0.25; SE=0.09; *P*=.005). There was also a significant simple main effect of time within the FM condition only, whereby acceptability increased from postprogram to the 1-month follow-up (MD=0.11; SE=0.04; *P*=.01). The main effect of group was not significant (*P*=.61), suggesting no differences in acceptability as a function of NSSI history.

When acceptability was assessed using the IMI, there were no significant interactions or main effects (all *P*>.05). Overall, results pertaining to acceptability were largely inconsistent with hypothesis 2. Differences did not emerge as a function of NSSI history, and IM was only rated as more acceptable than FM after the program when assessed using the TFA questionnaire, although this preference became nonsignificant by the 1-month follow-up time point.

## Discussion

### Overview

The aims of this study were to compare the effectiveness (objective 1) and acceptability (objective 2) of 4-week-long FM and IM instructional programs among university students with and those without a recent history of NSSI. It was hypothesized that all indices of effectiveness would significantly improve in response to the FM and IM conditions relative to the inactive control condition among all students (hypothesis 1a). In addition, among students with a recent history of NSSI, the IM program was expected to be more effective than the FM program (hypothesis 1b). Students with a recent history of NSSI were also expected to report greater acceptability in response to the IM program relative to the FM program (hypothesis 2).

### Effectiveness of FM Versus IM Programs

Overall, hypothesis 1a was partially supported. Some indices of effectiveness were significantly improved in the FM and IM conditions relative to the inactive control condition, whereas others were not significantly impacted. Specifically, overall dispositional mindfulness and 2 of its underlying facets (ie, nonjudging and describing), well-being, and psychological need satisfaction were all significantly higher in the FM and IM conditions relative to the inactive control condition both 1 week and 1 month after the program. These findings are consistent with previous research that has identified dispositional mindfulness, whether measured as a unidimensional construct or in terms of its facets, as a proximal outcome that is often one of the most strongly impacted within studies evaluating the effectiveness of MBP [9,30,67,68]. Previous studies have also demonstrated that the underlying facets of dispositional mindfulness often respond differentially to MBP [28,69,70], as was found in this study. Our results are also consistent with research demonstrating the benefits of MBP for the overall well-being of university students [6,8].

However, most of this previous research documented the effectiveness of MBP comprising both FM and IM instructions [6,8,67]. Our findings therefore build on these findings, suggesting that even when the elements of standard MBP (ie, FM and IM instructions) are parsed apart and taught independently of one another, their benefits for overall dispositional mindfulness (and particularly the facets of nonjudging and describing), well-being, and psychological need satisfaction may still be experienced. This is particularly promising in light of the growing evidence of the challenges associated with FM activities among university students with and those without a history of NSSI (eg, adherence and physical or psychological discomfort during practice [26,34]), which may undermine their regular use by underscoring the comparable effectiveness of regularly using IM activities. In addition, previous research on the positive relationship between mindfulness and psychological need satisfaction has been largely limited to cross-sectional studies [71,72]. Our findings extend the literature base by providing experimental evidence of the benefits of MBP for the psychological need satisfaction of university students.

While these findings were robust with Bonferroni correction in terms of overall dispositional mindfulness and psychological need satisfaction, when the correction was applied, only the FM program was favored over the control condition in terms of nonjudging, only the IM program was favored over the control condition in terms of describing, and all pairwise comparisons became nonsignificant in terms of well-being. As Bonferroni correction is highly conservative and its use is controversial [73,74], these discrepancies should be interpreted with caution. Nevertheless, they highlight the possibility that certain dispositional mindfulness facets (ie, nonjudging and describing) may be differentially impacted by the repeated practice of FM versus IM activities. Additional research in this area is therefore recommended to elucidate these findings.

Meanwhile, other facets of dispositional mindfulness (ie, awareness, nonreactivity, and observing), stress, emotion regulation styles, and academic engagement were not significantly impacted by university students’ participation in FM and IM programs. With regard to stress in particular, it is possible that university students’ resilience to stress improved as a result of their participation in the FM or IM program in this study (as evidenced by their improved overall dispositional mindfulness, well-being, psychological need satisfaction, etc), despite the degree of stress that they were perceiving remaining unchanged. Alternatively, for awareness, nonreactivity, stress, and academic engagement, the main effect of condition approached significance (with the *P*-value ranging from .05 to .08), suggesting that these nonsignificant findings may simply be attributable to a lack of statistical power. This is supported by the fact that previous studies have found FM and IM interventions to be effective at improving awareness [28,30,75], nonreactivity [28,30,76], and stress [6,7,77]. Although no previous studies have evaluated the impacts of MBP on academic engagement among university students, a meta-analysis of the impacts of mindfulness interventions on academic performance (a distinct but related construct) revealed significant positive effects among university students [78]. In terms of the dispositional mindfulness facet of observing, previous studies have noted mixed results related to the impacts of FM and IM interventions on observing. Some studies have found it to improve in response to these interventions [28,30,38,76], while others have found no impact on observing [75]. Additional research is therefore needed to clarify the precise conditions under which improvements in observing (ie, the tendency to pay attention to momentary sensory experiences) may be experienced in response to MBP.

Finally, this study was the first to explore the impacts of MBP on SDT-based emotion regulation styles (ie, integrative, suppressive, and dysregulated) and found that neither FM nor IM instructional programs impacted them. An individual’s emotion regulation style reflects their typical approach to interacting with their own anxiety and stress, that is, either by exploring and attempting to learn from those emotions (integrative), suppressing those emotions (suppressive), or feeling that they have no control over how those emotions are expressed (dysregulated) [79]. An integrative emotion regulation style has been shown to be positively associated with psychological well-being among adults with and those without a history of NSSI [79,80]. Thus, this study addressed the need to explore avenues for fostering an integrative approach to managing anxiety and stress (and simultaneously decreasing the use of suppressive or dysregulated approaches) among these populations. One possible explanation why the FM and IM instructional programs were not effective at fostering more adaptive emotion regulation styles among students may be a function of the university environment itself. Specifically, this environment is characterized by intense periods of stress and anxiety among students [1,2], and it is thus possible that the present programs were not sufficient to alleviate these pressures or students’ habitual approaches to interacting with them. A further investigation into other possible avenues for achieving adaptive emotion regulation styles in the university context is therefore needed.

Furthermore, hypothesis 1b was not supported. The effectiveness of the FM and IM programs did not differ as a function of NSSI history. Rather, the patterns of effectiveness held true for students with and those without a recent history of NSSI, refuting previous suggestions of a unique response of university students with a history of NSSI to FM activities, which was expected to arise as a result of their tendency to report elevated levels of emotion regulation difficulties [20], self-criticism [21], alexithymia [22], a complex relationship with their body [23,24], or a history of trauma [25]. Our findings are also consistent with the results of a recent study reporting that university students with and those without a recent history of NSSI responded similarly to FM and IM inductions (ie, brief, single-session practices of mindfulness [38]) and extend these results within the context of lengthier MBP. Overall, the benefits of FM and IM instructional programs, when delivered independently of one another, do not appear to be differentially incurred by university students with and those without a history of NSSI.

Taken together, our findings suggest that 4-week, online FM and IM instructional programs may not differ in their effectiveness at improving overall dispositional mindfulness, nonjudging, describing, well-being, and psychological need satisfaction among university students with and those without a recent history of NSSI. These benefits were also sustained 1 month following program completion. Given that IM is generally perceived as more feasible than FM to integrate into day-to-day life and often results in greater adherence to practice recommendations [37,81] and a greater intention to continue using the activities going forward [29], the present evidence that IM may be no less effective than FM is promising. Specifically, it suggests that explicitly teaching and emphasizing both FM and IM within university-based MBP that also includes relevant psychoeducational content may be an approach that is not only effective but also responsive to the diverse needs, preferences, and busy schedules of university students.

### Acceptability of FM Versus IM Programs

The second objective was to compare the acceptability of FM and IM instructional programs among university students with and those without a recent history of NSSI. This objective was motivated by emerging research suggesting that IM activities are more acceptable than FM activities among university students, especially those with a recent history of NSSI [26,38]. However, our findings with regard to acceptability did not differ as a function of NSSI history. Furthermore, the suggested preference for IM was only supported by 1 of our 2 measures of acceptability (ie, the TFA questionnaire) immediately after the program. When acceptability was assessed through the lens of SDT (ie, using the IMI), no differences between the FM and IM programs were found; both were rated as highly acceptable. Of note, half of the IMI items pertained to psychological need satisfaction (ie, perceived autonomy and competence) when engaging with the FM or IM activities, while the other half pertained to interest or enjoyment and value or usefulness (themes that are also present within the TFA questionnaire). It is possible that students’ perceived autonomy and competence were similar in response to both the FM and IM activities and that this, in part, drove the nonsignificant difference in acceptability between the FM and IM instructional programs when assessed using the IMI. This is further supported by the similar improvements in overall psychological need satisfaction in response to both programs in this study.

High acceptability was also reported in response to both the FM and IM instructional programs when assessed using the TFA questionnaire. However, there was an initial preference for the IM program after the program, which became nonsignificant in the month that followed program completion, due to satisfaction with the FM program significantly increasing during that time frame. Consistent with previous studies [82], the brevity and flexibility inherent in IM practice (and the perceived novelty of this approach for some students) may have been appealing to students from the outset, while regular FM practice may have initially been challenging to integrate into their busy routines. Over time, it is possible that students in the FM condition became more adept at integrating these activities into their day-to-day life and may have even begun noticing positive changes stemming from their practice. Indeed, findings from our preliminary analyses revealed that in the first 2 weeks of the FM and IM programs, those participating in the IM program were more likely to report frequent home practice relative to those participating in the FM program, but this discrepancy disappeared by the last 2 weeks of the programs. Thus, while initial reactions to FM versus IM may tend to favor IM [26,38], this discrepancy might diminish with repeated strategy use over time due to a gradual, increased appreciation of FM. In any case, on observing the postprogram mean levels of acceptability for FM versus IM when assessed using the TFA (mean_FM_=3.76; mean_IM_=4.03; possible range=1‐5) and the IMI (mean_FM_=5.03; mean_IM_=5.27; possible range=1‐7), it becomes evident that both the FM and IM instructional programs were initially perceived as highly acceptable in this study across both measures of acceptability and that these high ratings were either sustained or increased (in the case of FM) as time went on.

### Implications

Our findings have implications for both research and practice. First, they provide robust experimental evidence that, despite their unique experiences and coping needs, university students with a history of NSSI may not respond differently to FM and IM programs relative to students without such a history when it is delivered online and within the university context. Thus, future research may wish to focus more on potential nuances within these generally positive reactions, as it does not appear that FM is aversive for either group or that only students with a history of NSSI respond more favorably to IM than FM. For instance, future studies may wish to compare university students’ reactions to specific FM or IM activities, as well as the relative impacts of those activities on the different facets of dispositional mindfulness.

The findings also underscore the need for more research on the use of IM among student populations. First, the possibility of heterogeneity in responses to FM versus IM programs among other subgroups of university students (eg, those with neurodevelopmental or learning difficulties) should not be discounted; rather, additional research in this area is needed. Second, while this study contributes to a growing body of literature that documents the benefits of IM among university students [27,28,38], research exploring the use of IM among younger student populations, such as elementary and high school students, is scarce but shows preliminary evidence of the accessibility of IM among these youth [29]. As the cognitive processes of children and adolescents (eg, metacognition and attentional control) continue to develop [83,84], they may encounter challenges with FM activities, given that these activities require them to focus on their thoughts and emotions over an extended period of time. Thus, the brevity and flexibility of IM may be beneficial within elementary and high school contexts as well.

Finally, our results have important implications for the implementation of MBP on university campuses. Until now, regular FM practice has been emphasized as a precursor for achieving greater dispositional mindfulness [9-11] and, in turn, the broader mental health benefits of participating in MBP [9,29,30,64]. However, our results revealed that an IM instructional program was no less effective or acceptable than an FM instructional program at improving mental health and well-being outcomes, thereby unveiling an alternative approach to MBP in the university setting, which may be just as effective as the current standard approach. In recognition of students’ diverse needs, experiences, and preferences, it is therefore recommended that *both* FM and IM be equally emphasized and explicitly taught as part of MBP in the university context, where resources permit. This way, students may decide for themselves which approach is best suited to their individual needs. Alternatively, where funds and resources are limited, university student services may wish to focus their resources on the development and dissemination of IM programs, given that these may be initially perceived as the most highly acceptable among students, thereby supporting their potential for high uptake and impact. It may also be worth highlighting the accessibility and brevity of IM in communications to students about the benefits of MBP to support the uptake of such programming, as time restraints are a commonly cited reason for low engagement with MBP [85,86].

Moreover, our findings suggest that MBP offered to university students should include relevant psychoeducational content in addition to the instruction of FM and IM activities, as this was a foundational element of both programs in this study and may have contributed to their comparable effectiveness. Finally, the present FM and IM instructional programs were relatively brief (ie, 4 weeks) and were delivered online. As evidence of the effectiveness of digital MBP continues to grow [7,75,87], our findings contribute to this literature and suggest that the integration of similarly resource-efficient MBP in the university context may be a feasible way to enhance existing support to bolster students’ mental health and well-being.

### Limitations

The findings of this study must be considered within the context of this study’s limitations. First, the sample was disproportionately composed of women (80.6%), which is not representative of the general university student population or of the population of university students who report a history of NSSI [88,89]. This limits the generalizability of our findings and underscores the need for additional research that is more representative in terms of gender identity. Similarly, the findings may not be generalizable to clinical samples of young adults with a recent history of NSSI, who may experience higher levels of emotion dysregulation, self-criticism, low body regard, or alexithymia [20-24], or a more severe history of trauma [25] relative to a university sample, which may undermine the benefits of FM instruction in a more pronounced way [14]. Further research on the impacts of FM versus IM among clinical samples of young adults who report a recent history of NSSI is therefore needed. Finally, our analyses may have been slightly underpowered, as evidenced by many of our primary results approaching significance (ie, the *P* values pertaining to the main effect of condition for awareness, nonreacting, stress, and academic engagement ranged from .05 to .08). Additional studies with larger sample sizes are needed to ascertain the impacts of FM and IM instructional programs on these (and potentially other) outcomes among university students.

### Conclusion

Four-week FM and IM instructional programs were found to be effective at improving overall dispositional mindfulness, nonjudging, describing, well-being, and psychological need satisfaction among university students with and those without a recent history of NSSI, with sustained improvements over a 1-month period. Both programs were also found to be highly acceptable, although students noted a slight preference for IM immediately after the program. Notably, none of these results differed as a function of students’ NSSI history. The findings of this study underscore the effectiveness and acceptability of both approaches to MBP in the university context, as well as the potential value of explicitly teaching and emphasizing both FM and IM within university-based MBP, an approach that may be optimally responsive to diverse needs and preferences among students.

## Supplementary material

10.2196/70011 Multimedia Appendix 1 Formal and informal mindfulness program content.

10.2196/70011 Multimedia Appendix 2 CONSORT-EHEALTH checklist.

10.2196/70011 Multimedia Appendix 3 Results of 3-way analyses of covariance and ANOVAs across all outcomes.

## Abbreviations

ANCOVA: analysis of covariance
BPNSFS: Basic Psychological Need Satisfaction and Frustration Scale
ERI: Emotion Regulation Inventory
FFMQ: Five Facets of Mindfulness Questionnaire
FM: formal mindfulness
IM: informal mindfulness
IMI: Intrinsic Motivation Inventory
MBP: mindfulness-based programming
NSSI: nonsuicidal self-injury
PSS-10: 10-item Perceived Stress Scale
SDT: self-determination theory
TFA: Theoretical Framework of Acceptability
UWES-S: Utrecht Work Engagement Scale for Students
WEMWBS: Warwick-Edinburgh Mental Well-Being Scale
