# Supplementary material for: Web-Based Formal Versus Informal Mindfulness Programs for University Students With and Those Without Recent Self-Injury: Randomized Controlled Trial
Source: J Med Internet Res. 2025 Nov 27;27:e70011. doi: 10.2196/70011 (PMC12661606; doi:10.2196/70011)
Supplement: Multimedia Appendix 1 [file jmir-v27-e70011-s001.docx]

**Multimedia Appendix 1.** Formal and informal mindfulness program content.

| Session Number | Psychoeducational Content Taught | Strategies Taught (Formal) | Strategies Taught (Informal) |
| --- | --- | --- | --- |
| 1 | What is mindfulness?   - Formal vs. informal   How mindfulness works  Debunking mindfulness myths | Body scan | 1. Coming to your senses – basic  2. Anchoring with the breath  3. Mindful routine tasks |
| 2 | Stress  Emotion regulation   - Reacting vs. responding - Comfortable vs. uncomfortable emotions   Overcoming mindfulness hurdles | Sitting meditation | 1. Coming to your senses – advanced  2. Mindful meetings (sitting with uncomfortable emotions) |
| 3 | The chattering mind  Relationship between thoughts, emotions, and behaviour  Unhelpful thought patterns | Thought meditation | 1. Awareness of thoughts and re-anchoring |
| 4 | Self-criticism   - Functions and consequences   Self-compassion and mindfulness  Growth mindset and mindfulness | Loving-kindness meditation | 1. Daily loving-kindness  2. Responding to your inner critic  3. Random acts of kindness  4. Loving-kindness in conflict |
