# Supplementary material for: Web-Based Formal Versus Informal Mindfulness Programs for University Students With and Those Without Recent Self-Injury: Randomized Controlled Trial
Source: J Med Internet Res. 2025 Nov 27;27:e70011. doi: 10.2196/70011 (PMC12661606; doi:10.2196/70011)
Supplement: Multimedia Appendix 3 [file jmir-v27-e70011-s003.docx]

Multimedia Appendix 3

| **Table S2**  *Results of Three-Way ANCOVAs and ANOVAs Across All Outcomes* | | | |
| --- | --- | --- | --- |
| Outcome | Effect | Interaction/Main Effect Results | Pairwise Comparison Results |
| **Overall**  **Dispositional Mindfulness** | Condition*Group*Time | *F*(2, 205) = 0.59, *p* = .555, *η*_p_^2^ = .01 |  |
|  | Group*Time | *F*(1, 205) = 0.40, *p* = .530, *η*_p_^2^ = .00 |  |
|  | Condition*Time | *F*(2, 205) = 0.35, *p* = .703, *η*_p_^2^ = .00 |  |
|  | Group*Condition | *F*(2, 205) = 0.85, *p* = .430, *η*_p_^2^ = .01 |  |
|  | Group | *F*(1, 205) = 0.22, *p* = .641, *η*_p_^2^ = .00 |  |
|  | Condition | ***F*(2, 205) = 7.60, *p* < .001, *η*_p_^2^ = .07** | ***See Table 3 in main text*** |
|  | Time | *F*(1, 205) = 1.08, *p* = .300, *η*_p_^2^ = .01 |  |
| **Awareness** | Condition*Group*Time | *F*(2, 205) = 1.06, *p* = .347, *η*_p_^2^ = .01 |  |
|  | Group*Time | *F*(1, 205) = 0.63, *p* = .429, *η*_p_^2^ = .00 |  |
|  | Condition*Time | *F*(2, 205) = 1.34, *p* = .265, *η*_p_^2^ = .01 |  |
|  | Group*Condition | *F*(2, 205) = 0.61, *p* = .544, *η*_p_^2^ = .01 |  |
|  | Group | *F*(1, 205) = 1.34, *p* = .248, *η*_p_^2^ = .01 |  |
|  | Condition | *F*(2, 205) = 3.04, *p* = .050, *η*_p_^2^ = .03 |  |
|  | Time | *F*(1, 205) = 1.22, *p* = .270, *η*_p_^2^ = .01 |  |
| **Nonjudging** | Condition*Group*Time | *F*(2, 205) = 0.48, *p* = .622, *η*_p_^2^ = .01 |  |
|  | Group*Time | *F*(1, 205) = 0.28, *p* = .597, *η*_p_^2^ = .00 |  |
|  | Condition*Time | *F*(2, 205) = 0.51, *p* = .603, *η*_p_^2^ = .01 |  |
|  | Group*Condition | *F*(2, 205) = 1.59, *p* = .206, *η*_p_^2^ = .02 |  |
|  | Group | *F*(1, 205) = 0.37, *p* = .544, *η*_p_^2^ = .00 |  |
|  | Condition | ***F*(2, 205) = 3.75, *p* = .025, *η*_p_^2^ = .04** | ***See Table 3 in main text*** |
|  | Time | *F*(1, 205) = 1.12, *p* = .291, *η*_p_^2^ = .01 |  |
| **Nonreacting** | Condition*Group*Time | *F*(2, 205) = 0.42, *p* = .661, *η*_p_^2^ = .00 |  |
|  | Group*Time | *F*(1, 205) = 0.07, *p* = .799, *η*_p_^2^ = .00 |  |
|  | Condition*Time | *F*(2, 205) = 0.27, *p* = .761, *η*_p_^2^ = .00 |  |
|  | Group*Condition | *F*(2, 205) = 2.64, *p* = .074, *η*_p_^2^ = .03 |  |
|  | Group | *F*(1, 205) = 0.01, *p* = .927, *η*_p_^2^ = .00 |  |
|  | Condition | *F*(2, 205) = 2.53, *p* = .082, *η*_p_^2^ = .02 |  |
|  | Time | *F*(1, 205) = 0.21, *p* = .649, *η*_p_^2^ = .00 |  |
| **Observing** | Condition*Group*Time | *F*(2, 205) = 0.02, *p* = .981, *η*_p_^2^ = .00 |  |
|  | Group*Time | *F*(1, 205) = 0.00, *p* = .984, *η*_p_^2^ = .00 |  |
|  | Condition*Time | *F*(2, 205) = 0.38, *p* = .688, *η*_p_^2^ = .00 |  |
|  | Group*Condition | *F*(2, 205) = 1.58, *p* = .208, *η*_p_^2^ = .02 |  |
|  | Group | *F*(1, 205) = 0.11, *p* = .740, *η*_p_^2^ = .00 |  |
|  | Condition | *F*(2, 205) = 1.66, *p* = .194, *η*_p_^2^ = .02 |  |
|  | Time | *F*(1, 205) = 0.00, *p* = .952, *η*_p_^2^ = .00 |  |
| **Describing** | Condition*Group*Time | *F*(2, 205) = 0.98, *p* = .379, *η*_p_^2^ = .01 |  |
|  | Group*Time | *F*(1, 205) = 2.27, *p* = .133, *η*_p_^2^ = .01 |  |
|  | Condition*Time | *F*(2, 205) = 0.29, *p* = .746, *η*_p_^2^ = .00 |  |
|  | Group*Condition | *F*(2, 205) = 0.08, *p* = .925, *η*_p_^2^ = .00 |  |
|  | Group | *F*(1, 205) = 0.35, *p* = .553, *η*_p_^2^ = .00 |  |
|  | Condition | ***F*(2, 205) = 5.15, *p* = .007, *η*_p_^2^ = .05** | ***See Table 3 in main text*** |
|  | Time | *F*(1, 205) = 0.19, *p* = .665, *η*_p_^2^ = .001 |  |
| **Well-Being** | Condition*Group*Time | *F*(2, 209) = 0.64, *p* = .526, *η*_p_^2^ = .01 |  |
|  | Group*Time | *F*(1, 209) = 0.16, *p* = .691, *η*_p_^2^ = .00 |  |
|  | Condition*Time | *F*(2, 209) = 0.78, *p* = .460, *η*_p_^2^ = .01 |  |
|  | Group*Condition | *F*(2, 209) = 0.27, *p* = .768, *η*_p_^2^ = .00 |  |
|  | Group | ***F*(1, 209) = 10.81, *p* = .001, *η*_p_^2^ = .05** | **NSSI – No NSSI: *MD* = -3.16, *SE* = 0.96, *p* = .001** |
|  | Condition | ***F*(2, 209) = 3.22, *p* = .042, *η*_p_^2^ = .03** | ***See Table 3 in main text*** |
|  | Time | ***F*(1, 209) = 4.17, *p* = .042, *η*_p_^2^ = .02** | T3 – T2: *MD* = -0.14, *SE* = 0.52, *p* = .784 |
| **Stress** | Condition*Group*Time | *F*(2, 209) = 1.09, *p* = .340, *η*_p_^2^ = .01 |  |
|  | Group*Time | *F*(1, 209) = 1.44, *p* = .231, *η*_p_^2^ = .01 |  |
|  | Condition*Time | *F*(2, 209) = 0.84, *p* = .435, *η*_p_^2^ = .01 |  |
|  | Group*Condition | *F*(2, 209) = 1.80, *p* = .168, *η*_p_^2^ = .02 |  |
|  | Group | ***F*(1, 209) = 8.67, *p* = .004, *η*_p_^2^ = .04** | **NSSI – No NSSI: *MD* = 1.90, *SE* = 0.65, *p* = .004** |
|  | Condition | *F*(2, 209) = 2.60, *p* = .077, *η*_p_^2^ = .02 |  |
|  | Time | ***F*(1, 209) = 5.38, *p* = .021, *η*_p_^2^ = .03** | T3 – T2: *MD* = 0.14, *SE* = 0.69, *p* = .692 |
| **Psychological Need Satisfaction** | Condition*Group*Time | *F*(2, 207) = 0.87, *p* = .420, *η*_p_^2^ = .01 |  |
|  | Group*Time | *F*(1, 207) = 0.06, *p* = .805, *η*_p_^2^ = .00 |  |
|  | Condition*Time | *F*(2, 207) = 0.07, *p* = .933, *η*_p_^2^ = .00 |  |
|  | Group*Condition | *F*(2, 207) = 0.10, *p* = .906, *η*_p_^2^ = .00 |  |
|  | Group | ***F*(1, 207) = 5.70, *p* = .018, *η*_p_^2^ = .03** | **NSSI – No NSSI: *MD* = -0.14, *SE* = 0.06, *p* = .018** |
|  | Condition | ***F*(2, 207) = 5.43, *p* = .005, *η*_p_^2^ = .05** | ***See Table 3 in main text*** |
|  | Time | *F*(1, 207) = 1.78, *p* = .184, *η*_p_^2^ = .01 |  |
| **Integrative ER** | Condition*Group*Time | *F*(2, 208) = 2.55, *p* = .081, *η*_p_^2^ = .024 |  |
|  | Group*Time | ***F*(1, 208) = 4.93, *p* = .027, *η*_p_^2^ = .02** | ***See main text*** |
|  | Condition*Time | *F*(2, 208) = 0.26, *p* = .772, *η*_p_^2^ = .00 |  |
|  | Group*Condition | *F*(2, 208) = 0.10, *p* = .908, *η*_p_^2^ = .00 |  |
|  | Group | *F*(1, 208) = 1.25, *p* = .265, *η*_p_^2^ = .01 |  |
|  | Condition | *F*(2, 208) = 1.92, *p* = .149, *η*_p_^2^ = .02 |  |
|  | Time | *F*(1, 208) = 0.01, *p* = .945, *η*_p_^2^ = .00 |  |
| **Suppressive ER** | Condition*Group*Time | *F*(2, 208) = 0.13, *p* = .877, *η*_p_^2^ = .00 |  |
|  | Group*Time | *F*(1, 208) = 0.05, *p* = .827, *η*_p_^2^ = .00 |  |
|  | Condition*Time | *F*(2, 208) = 1.45, *p* = .237, *η*_p_^2^ = .01 |  |
|  | Group*Condition | *F*(2, 208) = 0.65, *p* = .523, *η*_p_^2^ = .01 |  |
|  | Group | *F*(1, 208) = 2.60, *p* = .109, *η*_p_^2^ = .01 |  |
|  | Condition | *F*(2, 208) = 0.10, *p* = .909, *η*_p_^2^ = .00 |  |
|  | Time | *F*(1, 208) = 1.61, *p* = .206, *η*_p_^2^ = .01 |  |
| **Dysregulated ER** | Condition*Group*Time | *F*(2, 208) = 0.86, *p* = .425, *η*_p_^2^ = .01 |  |
|  | Group*Time | *F*(1, 208) = 0.52, *p* = .473, *η*_p_^2^ = .00 |  |
|  | Condition*Time | *F*(2, 208) = 0.26, *p* = .771, *η*_p_^2^ = .00 |  |
|  | Group*Condition | *F*(2, 208) = 1.07, *p* = .346, *η*_p_^2^ = .01 |  |
|  | Group | *F*(1, 208) = 2.16, *p* = .143, *η*_p_^2^ = .01 |  |
|  | Condition | *F*(2, 208) = 0.72, *p* = .487, *η*_p_^2^ = .01 |  |
|  | Time | *F*(1, 208) = 2.66, *p* = .105, *η*_p_^2^ = .01 |  |
| **Academic Engagement** | Condition*Group*Time | *F*(2, 209) = 1.74, *p* = .178, *η*_p_^2^ = .02 |  |
|  | Group*Time | *F*(1, 209) = 1.93, *p* = .167, *η*_p_^2^ = .01 |  |
|  | Condition*Time | *F*(2, 209) = 0.49, *p* = .615, *η*_p_^2^ = .01 |  |
|  | Group*Condition | *F*(2, 209) = 0.44, *p* = .646, *η*_p_^2^ = .00 |  |
|  | Group | ***F*(1, 209) = 5.17, *p* = .024, *η*_p_^2^ = .02** | **NSSI – No NSSI: *MD* = -0.21, *SE* = 0.09, *p* = .024** |
|  | Condition | *F*(2, 209) = 2.78, *p* = .065, *η*_p_^2^ = .03 |  |
|  | Time | *F*(1, 209) = 0.75, *p* = .387, *η*_p_^2^ = .00 |  |
| **Acceptability (TFA)** | Condition*Group*Time | *F*(1, 141) = 0.00, *p* = .981, *η*_p_^2^ = .00 |  |
|  | Group*Time | *F*(1, 141) = 0.88, *p* = .350, *η*_p_^2^ = .01 |  |
|  | Condition*Time | ***F*(1, 141) = 5.70, *p* = .018, *η*_p_^2^ = .04** | ***See main text*** |
|  | Group*Condition | *F*(1, 141) = 1.10, *p* = .296, *η*_p_^2^ = .01 |  |
|  | Group | *F*(1, 141) = 0.27, *p* = .607, *η*_p_^2^ = .00 |  |
|  | Condition | ***F*(1, 141) = 4.66, *p* = .033, *η*_p_^2^ = .03** | **FM – IM: *MD* = -0.18, *SE* = 0.08, *p* = .033** |
|  | Time | *F*(1, 141) = 1.67, *p* = .198, *η*_p_^2^ = .01 |  |
| **Acceptability (IMI)** | Condition*Group*Time | *F*(1, 141) = 0.04, *p* = .845, *η*_p_^2^ = .00 |  |
|  | Group*Time | *F*(1, 141) = 0.05, *p* = .832, *η*_p_^2^ = .00 |  |
|  | Condition*Time | *F*(1, 141) = 0.28, *p* = .599, *η*_p_^2^ = .00 |  |
|  | Group*Condition | *F*(1, 141) = 0.15, *p* = .704, *η*_p_^2^ = .00 |  |
|  | Group | *F*(1, 141) = 0.83, *p* = .364, *η*_p_^2^ = .01 |  |
|  | Condition | *F*(1, 141) = 2.37, *p* = .126, *η*_p_^2^ = .02 |  |
|  | Time | *F*(1, 141) = 0.34, *p* = .561, *η*_p_^2^ = .00 |  |

*Note.* ER = Emotion regulation; TFA = Theoretical Framework of Acceptability questionnaire; IMI = Intrinsic Motivation Inventory; NSSI = nonsuicidal self-injury; T2 = post-program; T3 = 1-month follow-up. Bold text is used to highlight significant effects. Pairwise comparisons are reported without a Bonferroni correction; a positive mean difference (MD) favors the first group/time point noted.
